# Commercial Bank Credit Grading Model Using Genetic Optimization Neural Network and Cluster Analysis

**DOI:** 10.1155/2022/4796075

**Published:** 2022-05-31

**Authors:** Yunpu Bai, Dunlin Zha

**Affiliations:** School of Management, Nanjing University of Posts and Telecommunications, Nanjing 210003, China

## Abstract

Commercial banks are facing unprecedented credit risk challenges as the financial market becomes more volatile. Based on this, this study proposes and builds a credit risk assessment model for commercial banks based on GANN from the standpoint of commercial banks. In order to provide commercial banks with an effective and dependable credit risk assessment method, the indicators in this study are classified using cluster analysis, and then various representative indicators are chosen using a factor model, which takes into account the comprehensiveness of the information and reduces the complexity of the subsequent empirical analysis. On this basis, the network structure, learning parameters, and learning algorithm of commercial banks' credit risk assessment models are determined. Furthermore, advancements in data preprocessing and genetic operation have been made. According to simulation results, the highest accuracy rate of this method is 94.17 percent, which is higher than the BPNN algorithm 89.46 percent and the immune algorithm 90.14 percent. The optimization algorithm presented in this study improves the convergence speed and search efficiency of traditional algorithms, and the final experimental results show that the scheme is feasible and effective and can be used for commercial bank credit risk assessment.

## 1. Introduction

Commercial banks, as the financial institution with the most influence, the greatest number, and the broadest coverage in the financial market, play a variety of roles, including financing monetary capital, guiding capital flow, and regulating the balance of social supply and demand [1]. It holds a unique and crucial position in the entire financial system, as well as the national economy. At the moment, the rate of economic globalization is gradually accelerating, and financial market volatility is increasing by the day. Commercial banks face increasing financial risks as the “general hub” and “regulator” of a country's economic development [2]. Commercial banks' credit risk refers to the risk of economic losses caused by the customer's failure to perform the obligations in the agreed contract, that is, the possibility of the borrower defaulting due to the borrower's failure to repay the bank loan on time and in full for a variety of reasons [3]. Commercial banks' credit activities are divided into two categories: obtaining funds and using funds. If the risk of capital utilization is not effectively managed, the bank will face difficulties in obtaining funds, making it impossible for the bank to continue operations. Credit risk management is the most critical and difficult aspect of commercial banks' financial risk management. Effectively preventing and reducing credit risk has become the primary function of commercial bank operations and management. Simultaneously, in the context of interest rate marketization, commercial banks' pricing of loan interest rates is essentially the pricing of credit risk. As a result, credit risk is the most significant risk that commercial banks face. The ability to effectively control credit risk has become critical to commercial banks' profitability. Accurate and scientific credit risk evaluation is critical for providing a better basis for loan pricing. At present, most commercial banks, especially urban commercial banks with a short establishment time, mainly rely on manual work to complete credit risk assessment, which is unable to assess all enterprises with loan needs, and the assessment results are difficult to update in time. The development of ANN (artificial neural network) [4–6] and GAs (genetic algorithms) provides a new direction for credit risk analysis and evaluation of commercial banks.

ANN is a parallel distributed mode processing system developed by applying mathematical methods based on the research results of neuropsychology and cognitive science. It has high parallel computing ability, self-study ability, and fault tolerance. The basic constituent elements of ANN are neurons, which are connected together in the form of nodes through some mode, and nodes can communicate with each other. The output information will be transmitted to other nodes through the connection right, which can inhibit or excite the information being transmitted, just like a real neuron. ANN can carry out complex logic operations and deal with nonlinear problems and abstract, simplify, and simulate biological NN (neural network). ANN is not strict about the distribution of data, and it is not necessary to describe the functional relationship between independent variables and dependent variables in detail. These characteristics make it a hotspot of credit risk analysis methods. Although NN has been widely used, it also has its own shortcomings, which are mainly reflected in the uncertainty of the training process. The emergence of GA makes the training of NN have a new look. Using GA instead of NN algorithm to search the connection weight of NN can solve the problem of NN falling into the local minimum. Therefore, this study constructs a credit risk assessment model for commercial banks based on GANN (genetic algorithm neural network). The specific innovations are as follows:

1. Aiming at the shortcomings of BPNN (backpropagation neural network), such as easy to fall into the local minimum and slow convergence speed, this study introduces GA to design a practical GA coding scheme. In addition, reasonable fitness function, crossover operator, and mutation operator are used in genetic operation to optimize BPNN.
2. This study establishes a credit risk evaluation index system based on industry classification, with quantitative indicators as the main factor and qualitative indicators as the auxiliary factor. And the improved technology and method of credit index data preprocessing are given. On the basis of discussing the related theories of credit risk assessment of commercial banks, this study focuses on the in-depth study of credit risk assessment methods and makes empirical analysis by using the constructed model and evaluation index system to verify the rationality and feasibility of the evaluation system constructed in this study.

The study is organized as follows: Section 1 introduces the background and significance of the topic selection and puts forward the research innovation and structural arrangement of this study. Section 2 describes the related works and analyzes the research status of NN and commercial bank credit risk at home and abroad and gives the research content and work of this study. Section 3 summarizes the relevant theoretical basis, constructs the bank credit risk assessment model based on GANN, and introduces its software implementation method in detail. Section 4 makes an empirical analysis of the index system and the improved GANN model, verifies the scientificity and rationality of the evaluation system, and shows that the algorithm model constructed in this study has certain practical value. Section 5 summarizes the conclusions and limitations of the full-text research and puts forward the future research direction.

## 2. Related Works

With the advancement of global economic integration, financial security has become a global concern. How to achieve financial security and how to ensure commercial banks' credit risk management are also common issues discussed by countries all over the world. Many aspects of ANN make it a popular credit risk analysis method. In recent years, with the advancement of information technology, an artificial intelligence method [7–9] with machine learning [10] capability has been introduced into credit risk assessment. The main goal of this stage is to develop a quantitative credit risk assessment model.

Cole and others studied the credit risk assessment of commercial banks by using genetic programming method and tested the model empirically by using the actual data of commercial banks in China. The test results show that this model is superior to a traditional statistical model, NN model, and decision tree model in prediction accuracy, practical value, and robustness. Masoudik et al. analyzed the microscopic and easily quantified non-systematic risks in the financial risks of commercial banks. On the basis of identifying types and root causes, referring to international practices and international financial regulations, and combining with the reality of Chinese commercial banks, this study systematically designs the non-systematic risk monitoring and early warning index system and corresponding early warning signals of commercial banks [11]. Eckert et al. and others can make efficient and timely judgments by introducing domain knowledge and using specific artificial intelligence methods such as GA, NN, and decision tree and provide effective decision support for decision makers [12]. The research of Yu et al. and others showed that under the same data environment, the accuracy of credit risk assessment by NN is higher than other assessment methods [13]. Huang et al. compared the early warning model based on ANN with the early warning model based on probability and reduced the rate of occurrence errors by reconstructing the data set. The results show that the NN model is superior to the probabilistic model in discrimination accuracy [14]. Soui et al. and others discussed and compared the application of the expert system in the field of credit risk analysis from the perspective of knowledge acquisition [15]. Labriola used a fixed-length code to represent the risk identification criteria and solved the credit risk assessment problem by using GA [16]. Plosser and Santos and others thought that when using GA to solve practical problems, operators need to have a deep knowledge of algorithms and a comprehensive understanding and good judgment of the problem itself, and the limitations of genetic coding itself hinder the application of GA to a certain extent [17]. Zhang et al. and others used NN to predict the failure of a company, and the results showed that NN-like prediction had better prediction ability than discriminant analysis [18]. Zhang et al. and others suggested that GA should be used to screen the input indicators and combine them with ANN to work together. They used this method to analyze the data of bankrupt enterprises 3 years before the bankruptcy, and the results showed that the discrimination accuracy of ANN network model was much better than that of comparison model [19]. Wang et al. and others used a rough set and NN method to predict credit risk, and the rough set was used to screen financial variables, thus improving the explanatory ability of the NN method, and the prediction accuracy was correspondingly improved [20]. Based on the research of previous relevant literature and GANN, this study puts forward and constructs the credit risk evaluation model for commercial banks. Aiming at the shortcomings of BPNN, such as easy to fall into the local minimum and slow convergence speed, GA is introduced to design a practical GA coding scheme. A credit risk assessment index system classified by industry, dominated by quantitative indicators, and supplemented by qualitative indicators has been established. It also gives the improved technology and method of credit index data preprocessing. In genetic operation, more reasonable fitness function, crossover operator, and mutation operator are used to optimize BPNN. Experiments verify that the evaluation system constructed in this study has certain accuracy and feasibility.

## 3. Methodology

### 3.1. GANN

The three elements of ANN are (1) synapse or connection weight: the connection degree between different neurons is given by the connection weight, negative value indicates inhibition and positive value indicates excitation; (2) summation unit or adder: similar to linear combiner, it obtains the weighted sum of input information; and (3) activation function or transfer function: nonlinear mapping, which can distribute the output value within a certain range, such as [0, 1] or [−1, 1]. As a powerful tool to study complexity, the ANN method has shown its unique advantages in pattern recognition and classification, automatic control, financial distress prediction, etc. It can deal with a series of ratio type information input, and then find its law from a large number of complex data of unknown mode through continuous learning and produce corresponding output, so as to generate a mode that successfully reflects the corresponding relationship between input and output variables. According to the information flow and network topology, the ANN model can be divided into feedforward network and feedback network. Each layer of neurons in feedforward NN receives input information from the previous layer and transmits it to the next layer. There is only one direction and there is no feedback of information. It is the most common and easy to program network structure. A feedforward network includes two basic forms: single-layer feedforward network and multilayer feedforward NN [21]. A single-layer feedforward network consists of an input layer and an output layer. There is no hidden layer in the middle. It can only solve the classification problem of linear separability. A multilayer feedforward NN is composed of an input layer, several hidden layers, and an output layer. It can be used to solve nonlinear classification problems. A single-layer feedforward network is the simplest layered network, whereas a multilayer feedforward network is the most widely used network structure in NN. In the feedback NN, the output information of some neurons is fed back to the upper layer or the same layer, and the information flow is interleaved in the forward and reverse directions.

The role of ANN in credit risk analysis is carried out through the classification function of ANN [22]. That is to say, firstly, a group of factors that affect the classification is found, which are used as the input of ANN, and then an ANN credit risk analysis model is formed through training with or without tutors. For the new sample input, this model can produce the discrimination of credit risk. The NN model includes two processes: training and testing. Firstly, the neurons are trained with training set data, so that different input vectors can get corresponding output values. By repeatedly adjusting and modifying the parameters and thresholds until the error is within the specified range, the training process of NN is completed. The test process is to input a group of data of non-training indicators into the model generated in the training process to test the accuracy of model classification. The ANN method overcomes the complexity of traditional analysis process, especially the establishment of model function and brings great convenience to modeling and analysis. BPNN is a kind of multilayer feedforward NN. BPNN is considered to be the most suitable approximate relationship for analog input and output. It is one of the most widely used ANNs with mature algorithm. BPNN is a multilayer feedforward NN according to the backward propagation of errors, and its learning process consists of forward propagation of signals and backward propagation of errors. Although BPNN has been widely used, it also has its own shortcomings, which are mainly manifested in the uncertainty of the training process.

GA is a relatively new and effective optimization technology. GA is the most well-known evolutionary algorithm at the moment, and it is widely used in machine learning, engineering technology, data mining, computer science, pattern recognition, image processing, social science, and other fields. GA is a global random search algorithm that solves complex problems by simulating the genetic and long-term evolution processes of biology. GA is a group optimization algorithm with the ability to perform global searches due to its multipoint search, allowing the search results to avoid converging to the local optimal solution. As a result, the global optimal solution is obtained, and the accuracy of network evaluation is improved. Some criteria can be used to determine the termination condition of GA, and the algorithm's running process can be terminated when it is determined that the population has matured and there is no evolutionary trend. There are two commonly used criteria: (1) reaching the preset number of iterations and (2) the difference of average fitness of successive N generations of individuals is less than a certain minimum threshold. The evolutionary nature of GA makes it possible to search for excellent structure in parallel, thus reducing the possibility of falling into local optimum, and this method is also sensitive to the change of credit index. The emergence of GA makes the training of NN have a brand-new look. Using GA instead of BP algorithm to search the connection weight of NN is expected to solve the problem that BPNN falls into the local minimum.

### 3.2. Credit Risk of Commercial Banks

Credit risk can be defined in two ways: broadly and narrowly. Generalized credit risk refers to the uncertainty or volatility of the future impact of various uncertain factors on commercial banks, such that actual income deviates from expected income, suffers losses, or gains additional income. Narrow credit risk perception means that in the credit business, if the debtor or borrower fails to perform the contract and repay the loan principal and interest, the creditor or bank will incur financial losses because it will not receive the expected benefits. Commercial banks are credit-based industries with high debt and risk, with the profit point of settlement business and money management lending. Commercial banks' operating characteristics, as well as their important position and key role in maintaining national economic stability, have resulted in the characteristics of concealment and diffusion of bank operating risks. Credit risk arises from the asymmetry of information between the two parties to the transaction. This phenomenon occurs before the two parties sign the contract, which is referred to as asymmetric information beforehand, and it is easy to produce adverse selection. Customer credit risk has a direct impact on the quality of bank credit assets, and it can even lead to bank bankruptcy.

**Figure 1:**
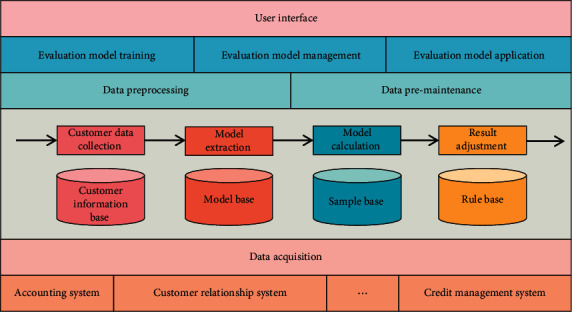
Overall structure of the credit risk assessment model for commercial banks.

Credit risk management process is the concrete implementation of credit risk management policy, the refinement and embodiment of credit risk management, and the main content of credit risk management. Credit management reflects the core competitiveness of commercial banks, and directly or indirectly affects the quality of bank assets, asset growth, income level, and financing strategy. The main goal of credit management is to evaluate the credit risk so as to effectively supervise and control the risk. In the transaction activities in the financial market, for commercial banks, there are intricate credit and interest relationships with borrowers, and the interrelated borrower enterprises form a credit chain. Once an enterprise in the credit chain defaults and fails to repay its accounts payable on schedule, it will cause a series of enterprises in the credit chain to default. Credit risk and credit risk for commercial banks, their subjects are the same, and all of them are due to the change of the debtor's credit status, which leads to the emergence of the value risk of bank credit assets. The difference lies in the range of financial assets they cover. The general framework of the commercial bank credit risk assessment model is shown in Figure 1.

Commercial banks develop relevant policies in order to create an efficient risk management organizational structure and to identify, analyze, evaluate, and respond to credit risks. Credit risk is an unavoidable risk in the day-to-day operations of commercial banks because banks themselves are in high-risk industries, and the asset market is volatile. The goal of improving credit risk management is to reduce the likelihood of such losses occurring. Credit risk assessment refers to the qualitative analysis and quantitative calculation of credit risk influencing factors using appropriate technical means, as well as the assessment and calculation of the borrower's default probability, in order to provide a decision-making basis for commercial banks' credit activities. The overall framework of the credit risk management system primarily consists of the organizational structure, management process, internal control, and credit risk management information reporting and disclosure. After nearly a century of evolution, the basic methods of commercial bank credit risk evaluation have progressed from simple to complex, including proportional analysis, statistical analysis, and artificial intelligence analysis. The methods used in the proportional analysis and statistical analysis stages, for example, can also be referred to as traditional credit risk assessment methods. Despite the fact that the traditional credit risk assessment method has significant limitations, it serves as a valuable reference for modern credit risk assessment.

### 3.3. Construction of Bank Credit Risk Evaluation Model Based on GANN

**Figure 2:**
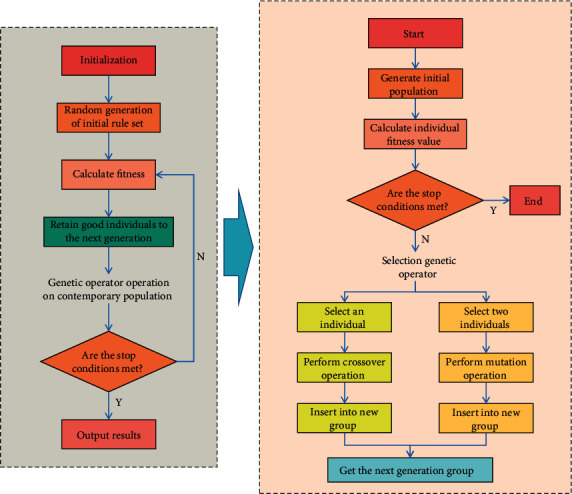
Genetic algorithm optimization process.

In this section, GA is used to optimize BPNN, which is easy to fall into the local minimum and slow in convergence. Before modeling, the data should be standardized to eliminate the influence of different dimensions and improve the accuracy of the model. The basic idea of the optimization algorithm in this study is: firstly, GA is used to globally optimize the weights and thresholds of NN, and then BPNN is used to accurately solve the problem, so as to realize complementary advantages and better solve the problem. Because the system is always developing and changing, the selected learning samples should not only reflect the characteristics of the system when it develops steadily, but also reflect the characteristics of the system when it suddenly changes, and at the same time, take into account all stages of the system development. According to the differences of financial indicators of different enterprises, this study divides the samples into different sets according to the comparability and similarity of financial characteristics of enterprises, and each such set is called a model. The division of patterns is related to the financial characteristics of enterprises and is limited by the number of valid samples under this pattern. If the number of pattern samples meets the requirements of building an evaluation model, the pattern is called an effective pattern; otherwise, it is called an invalid pattern. When establishing the index system, this study selects 18 financial and nonfinancial secondary indicators reflecting the status of loan enterprises. According to whether these indicators have significant differences between normal loans and loss loans, it is determined whether the indicators can be used as input index vectors. This study constructs the index system and follows the following basic principles: scientificity, comprehensiveness, pertinence, and applicability. Considering the availability of data, it constructs a credit risk evaluation index system suitable for commercial banks. The flow of genetic algorithm optimization is shown in Figure 2.

The article chooses three-layer BPNN structure. The input layer contains 19 input units, the hidden layer has 10 nodes, and the output layer contains 4 output units. Since the input initial values and initial weights have a great relationship with whether the learning can reach the local minimum and converge, it is necessary to preprocess the input initial values, that is, normalize them. For the continuous data in qualitative indicators, the feature discretization technology can be used for reduction. The advantage of this technology is that it simplifies the data description, not only greatly reduces the complexity of data mining calculation, but also makes the relationship between data and the final data mining results easier to understand. Data discretization is the process of merging attribute values. By scientifically merging attribute values, the number of attribute values is reduced, thus reducing the complexity of the problem. It is beneficial to improve the classification performance of rule sets obtained in the process of knowledge learning. Generally, when determining the number of neurons in the hidden layer, it is usually necessary to satisfy the following two formulas:

(1) $$\begin{array}{c} m=\sqrt{x+y}+R\left(10\right),\\ 2^{m}>y, \end{array}$$

where *m* is the number of neurons in the hidden layer, *x* is the number of neurons in the output layer, and *y* is the number of neurons in the input layer. *R*(10) represents any integer between 1 and 10. Assuming that the set *X*={*U*_2_, *U*_3_, *U*_4_, *U*_5_} and the conditional attribute set C={A_1_,A_2_}, the roughness calculation process of the set *X* is as follows:

(2) $$\begin{array}{c} R^{-}\left(X\right)=\left\{x\in U|R\left(x\right)\subseteq X\right\}=\left\{U_{2},U_{3},U_{4},U_{5},U_{7}\right\},\\ R_{-}\left(X\right)=\left\{x\in U|R\left(x\right)\cap X\neq 0\right\}=\left\{U_{2},U_{4},U_{5}\right\}\neq \emptyset . \end{array}$$

Therefore:

(3) $$\begin{array}{c} \rho \left(X\right)=1-\frac{\left|POS_{C}\left(X\right)\right|}{\left|R^{-}\left(X\right)\right|}=0.6. \end{array}$$

If *X*={*U*_2_, *U*_3_}, it is not definable because:

(4) $$\begin{array}{c} R^{-}\left(X\right)=\left\{x\in U|R\left(x\right)\subseteq X\right\}=\left\{U_{2},U_{3},U_{5},U_{7}\right\},\\ R_{-}\left(X\right)=\left\{x\in U|R\left(x\right)\cap X\neq \emptyset \right\}\neq \emptyset . \end{array}$$

Because there are redundant attributes and noise data in training data, it will affect the efficiency and convergence speed of GA. Therefore, attribute reduction of raw data is needed to improve the algorithm efficiency. This study uses the attribute reduction algorithm based on attribute importance. This method can accurately reduce redundant attributes and maintain the original correlation between data, which provides a strong guarantee for the performance and accuracy of GA. The output layer of this study adopts a neuron, and the output value is close to indicate that the enterprise is classified as a high-risk category, and the closer the output value is, it indicates that the enterprise belongs to a low-risk category. Suppose that in the *q*th iteration, the output error *p* of the *e*_*p*_(*q*)th node at the output end:

(5) $$\begin{array}{c} e_{p}\left(q\right)=t_{p}\left(q\right)-y_{p}\left(q\right),\\ p=1,2,\ldots ,k, \end{array}$$

where *t*_*p*_(*q*) is the target output value, *y*_*p*_(*q*) is the actual output value, and the mean square error signal is:

(6) $$\begin{array}{c} \xi_{p}\left(q\right)=\frac{1}{M}\sum_{s=1}^{M}\left(\frac{1}{k}\sum_{l=1}^{k}e_{p}{\left(q\right)}^{2}\right)=\frac{1}{M}\sum_{s=1}^{M}\left(\frac{1}{k}\sum_{l=1}^{k}{\left(t_{p}\left(q\right)-y_{p}\left(q\right)\right)}^{2}\right). \end{array}$$

The above formula can be expressed as a vector:

(7) $$\begin{array}{c} \xi_{p}\left(q\right)=\frac{1}{M}\sum_{s=1}^{M}\left(\frac{1}{k}\sum_{l=1}^{k}{\left(T\left(q\right)-Y\left(q\right)\right)}^{2}\right). \end{array}$$

The gradient of *ξ*(*q*) with respect to *v*(*q*) is:

(8) $$\begin{array}{c} \nabla \left(\xi \left(q\right)\right)=\frac{\partial \xi \left(q\right)}{\partial v\left(q\right)}\\ =\frac{\partial \xi \left(q\right)}{\partial Y\left(q\right)}\cdot \frac{\partial Y\left(q\right)}{\partial v\left(q\right)}\\ =-E\left(q\right)g\left(U\left(q\right)\right){\left[g\left(W{\left(q\right)}^{T}\right)X+B_{1}{\left(q\right)}^{T}\right]}^{T}. \end{array}$$

The initial parameters are set as follows: initial threshold *θ*, initial weight *W*, and momentum term *α*. The values of *Z*_*j*_ and *y*_*k*_ are calculated according to the following formulas:

(9) $$\begin{array}{c} z_{j}=f\left(\sum_{i=1}^{n}w_{jk}x_{i}-\theta_{j}\right),\\ y_{k}=f\left(\sum_{j=1}^{l}w_{jk}z_{j}-\theta_{k}\right), \end{array}$$

where *n* is the number of samples, *θ*_*i*_ is the threshold between the hidden layer and the input layer, *θ*_*k*_ is the threshold between the output layer and the hidden layer, and *l* is the number of neurons in the hidden layer. The error between the layers is found according to the following formula:

(10) $$\begin{array}{c} \delta_{jk}^{\phi }=\left(t_{l}^{\phi }-y_{l}^{\phi }\right)y_{l}^{\phi }\left(1-y_{l}^{\phi }\right),\\ \delta_{ij}^{\phi }=z_{j}^{\phi }\left(1-z_{j}^{\phi }\right)\sum_{k=0}^{m}\delta_{jk}^{\phi }w_{jk}, \end{array}$$

where *m* is the number of neurons in the output layer.

When using GANN model for credit risk assessment and analysis, we should first set the corresponding parameters of NN to ensure the effective operation of the assessment model. Then the credit risk evaluation index value and target value of the evaluation sample are inputted, and the network to make the output value close to the target value is trained. In this study, in the process of optimizing BPNN by GA, the main functions and parameters of GA are: determining 800 generations of genetic algebra. Using real coding method, the fitness function is selected according to the error. The larger the error, the smaller the fitness. The selection is based on the fitness scale method. Single point crossing, the crossing rate is 1. Uniform variation, the variation rate is 0.08. The fitness function will directly affect the efficiency of solving problems with GA. A good evaluation function should better reflect the background knowledge of solving the problem, which is more conducive to guiding GA to search for a better solution space. In this study, every training data are matched with every rule of the solution. If any rule matches the training data, it is considered as a correct classification of the solution. The fitness of each individual can be defined as the square of the ratio of the number of times it correctly classifies training data to all training data. Learning rate is the magnitude of weight adjustment after each step of training. The greater the learning rate, the greater the magnitude of weight adjustment and the faster the error curve converges. The gradient descent method used in NN operation may cause the error plane to fall into the local minimum, and the additional momentum model constructed in this study can effectively solve this problem.

## 4. Result Analysis and Discussion

In the simulation experiment, the number of nodes in the input layer is determined by the number of sample indexes. Different number of hidden layer nodes will form different NN models, which need constant and repeated debugging to obtain appropriate network parameters, thus forming an effective credit risk assessment network model. In this study, the company that has been specially treated is called ST company, and the company that has not been specially treated is called non-ST company. It means that the company's credit risk is relatively poor, which means that the company has fallen into debt repayment or has a high possibility of credit risk. Non-ST company means that the company operates normally and the possibility of default is very small. It is used to represent the sample with good credit. ST is used to represent the default sample, and the output value in the model is represented by 1. The output value of non-ST company in the model is represented by 0. The number of output layer nodes is 1. With 0.5 as the critical point, when the output value is less than 0.5 and close to 0, it belongs to ST enterprise. When the output value is greater than 0.5 and close to 1, it is a non-ST enterprise.

This study selects 350 listed companies as test samples, including 56 ST companies and 294 non-ST companies. After the data of 350 companies in the test sample are standardized, the value of credit risk evaluation index can be used as the input mode. The 350 companies selected in this study cover the electronics industry, chemical industry, paper industry, wine industry, chemical fiber industry, science and technology industry, pharmaceutical industry, automobile manufacturing, and other industries, which are quite representative. The specific settings are shown in Table 1.

**Figure 3:**
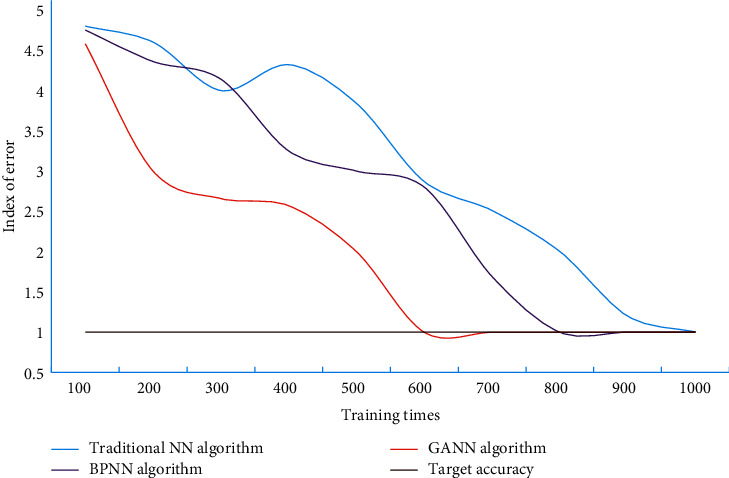
Training of different models.

This study uses MATLAB tools to build GANN, and then uses VisualC++, which can develop applications with good interactive functions, compatibility, and expansibility, to write user interface programs. In the standardized test sample input, the network model is used to simulate the operation, without setting the target expectation value, and the output value of the model is compared with the sum to check the accuracy of the simulation. The function of gradient descent backpropagation algorithm with adaptive adjustment of learning rate and momentum factor is a combined optimization algorithm of gradient descent method and adaptive adjustment of learning rate method. In this study, the training function chooses traingdx algorithm.  Figure 3 shows the training of different models.

It can be seen from the Figure 3 that the traditional NN algorithm evaluation model took 912 steps to achieve the accuracy requirement, the BPNN algorithm evaluation model took 742 steps to achieve the accuracy goal, and the improved BP algorithm evaluation model only took 523 steps to achieve the accuracy goal.

**Figure 4:**
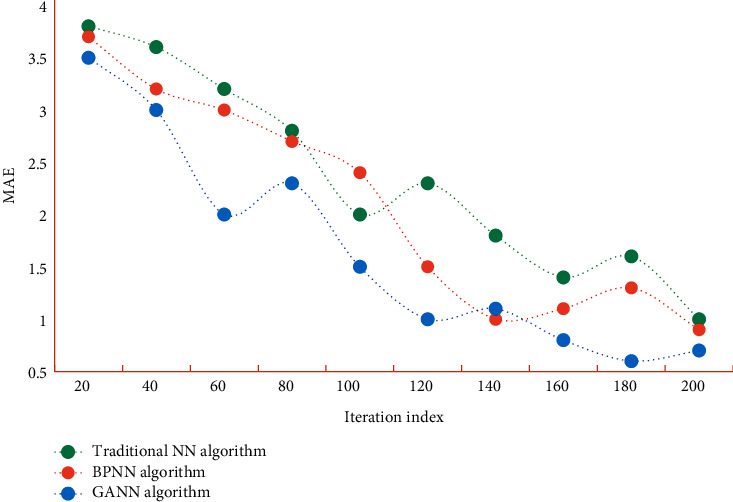
MAE of different algorithms on data set A.

**Figure 5:**
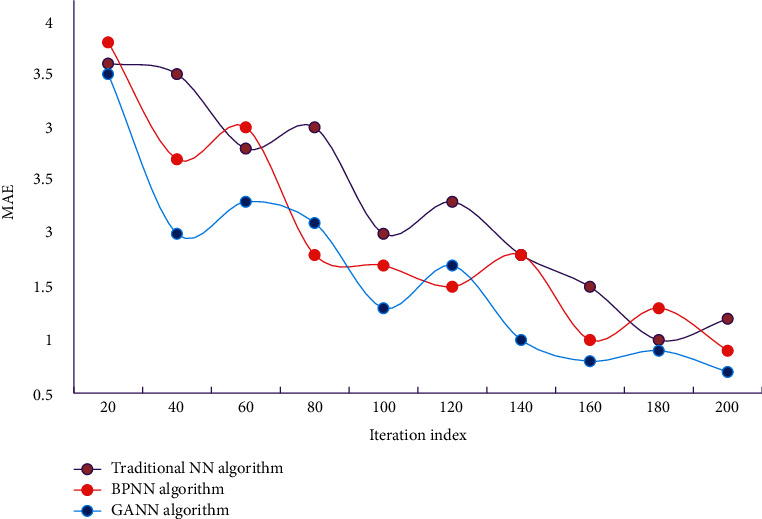
MAE of different algorithms on data set B.

In order to further illustrate the accuracy and reliability of the model constructed above, we also need to verify it with test samples. The training errors of traditional NN algorithm, BPNN algorithm, and GANN algorithm on data set A are shown in Figure 4. The mean absolute error of traditional NN algorithm, BPNN algorithm, and GANN algorithm on data set B is shown in Figure 5.

It can be seen from Figures 4 and 5 that the MAE values of GANN algorithm on different data sets are all at a low level. Compared with the contrast algorithm, the error is smaller, which shows that this algorithm has certain accuracy. In this study, the initial weight, learned weight, training data set, test data set, application data set, and other data are established into files to facilitate program call. The performance comparison of immune algorithm, traditional NN algorithm, BPNN algorithm, and GANN is shown in Table 2.

**Figure 6:**
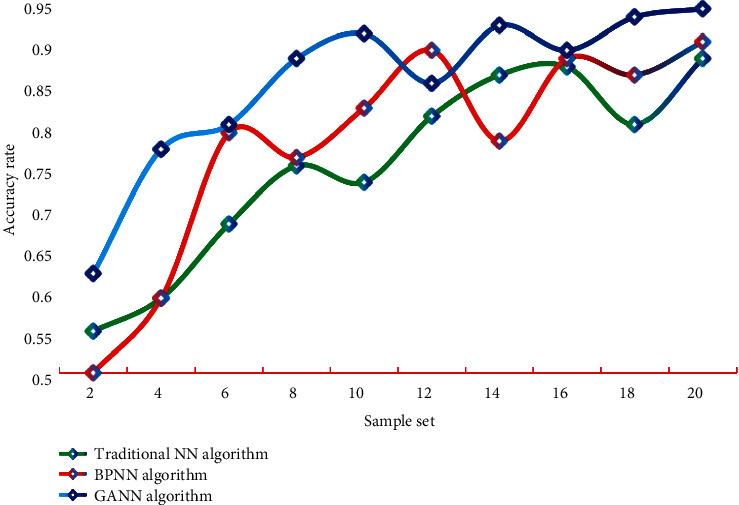
Accuracy performance of different algorithms on data set A.

**Figure 7:**
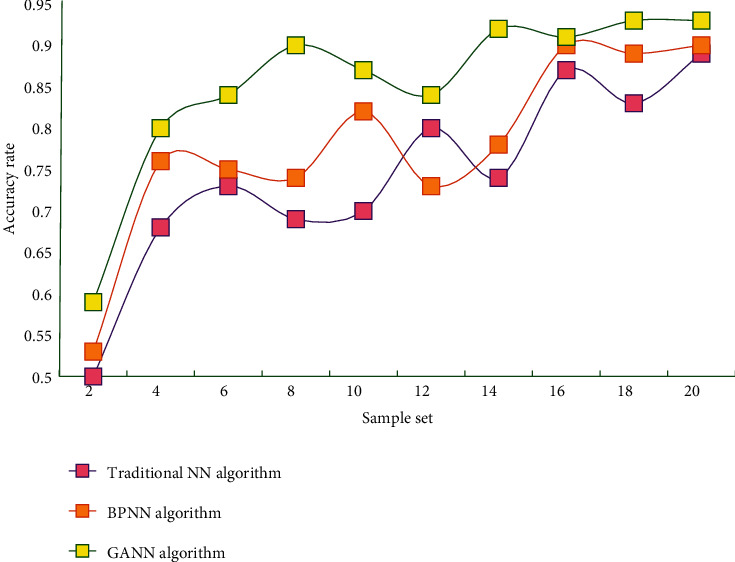
Accuracy performance of different algorithms on data set B.

From the data in the table, it can be seen that BPNN with GA optimized weights has greater advantages in time and accuracy than other three different models. It can be seen that GANN has strong advantages in evaluating the credit risk of commercial banks. In order to ensure the reliability and credibility of the obtained results, this study draws the accuracy performance of different algorithms on different data sets into data graphs, as shown in Figures 6 and 7.

It can be seen that the accuracy of this method is always at a high level, whether on data set A or B. The highest accuracy rate of this method is 94.17%, which is higher than that of BPNN algorithm 89.46% and immune algorithm 90.14%. The accuracy of GANN in this study is better than the other two algorithms, and this result shows the accuracy and reliability of the optimization algorithm in this study. It can be seen that the model constructed in this study has certain practicability and practical significance.

## 5. Conclusions

Commercial banks play an important role in the implementation of financial policies and social investments. Credit risk identification, evaluation, monitoring, reporting, and control are the main components of commercial banks' credit management. The assessment of credit risk is the cornerstone of all of them. This study builds a credit risk assessment model for commercial banks using GANN based on credit risk research. In this model, GA is used to create a practical GA coding scheme that addresses the shortcomings of BPNN, such as its tendency to fall into the local minima and slow convergence speed. In addition, a credit risk evaluation index system is established, which is classified by industry and is primarily based on quantitative indicators, with qualitative indicators supplemented. In addition, improved credit index data preprocessing technology and methods are presented. According to simulation results, this method has a higher accuracy rate of 94.17 percent than the BPNN algorithm (89.46 percent) and the immune algorithm (90.14 percent). Traditional algorithms' convergence speed and search efficiency are improved by the optimization algorithm presented in this study. It has some practical value and significance, and it provides a method for commercial banks to assess credit risk that is both effective and reliable. However, due to time and level constraints, some parts of this study continue to have flaws: the improvement in the fitness function and the correlation analysis of credit risk assessment indicators are insufficient. The following step in this study will optimize the improvement in the fitness function and investigate the correlation of credit risk assessment indicators in depth.

## Figures and Tables

**Table 1:** Performance comparison of different algorithms.

Industry	ST company	Non-ST company
Electronic industry	39	8
Chemical industry	51	4
Paper industry	28	7
Brewing industry	17	10
Chemical fiber industry	45	6
Technology industry	42	4
Pharmaceutical industry	26	5
Automobile manufacturing industry	34	9
Other industries	12	3
Total	294	56

**Table 2:** Performance comparison of different algorithms.

Algorithm	Accuracy (%)	Training time
Immune algorithm	90.14	7.412
Traditional NN algorithm	86.73	8.293
BPNN algorithm	89.46	7.014
GANN algorithm	94.17	6.154

## Data Availability

The data used to support the findings of this study are available from the corresponding author upon request.
